# The Long Fox Memorial Lecture: The Reality of Delusions

**Published:** 1928

**Authors:** Henry Devine

**Affiliations:** Medical Superintendent of Holloway Sanatorium, Virginia Water, Surrey


### THE LONG FOX MEMORIAL LECTURE:

DELIVERED IN THE UNIVERSITY OF BRISTOL ON NOVEMBER 29TH, 1927.

THE VICE-CHANCELLOR (Mr. T. LOVEDAY, M.A.) *in the Chair.*

BY

HENRY DEVINE, O.B.E., M.D., B.S., F.R.C.P.,

*Medical Superintendent of Holloway Sanatorium, Virginia Water, Surrey;*

ON

### THE REALITY OF DELUSIONS.

---

My first duty is to thank those who have invited me to deliver this the sixteenth Long Fox Memorial Lecture. I am unable to conceive of any greater honour which could be conferred upon one who was educated at the Bristol Medical School than that of being deemed worthy, by those who were his teachers and fellow-students, to give the address in which that great and good Bristol physician, Edward Long Fox, is annually commemorated.

I have selected "Delusions" as the subject of this lecture for two reasons. The first is as follows: delusions being the most prominent, frequent and characteristic symptoms in the psychoses, it is evident that the more insight we gain in respect of their origin and significance the nearer shall we be to solving the obscure and elusive problems of mental illness. The second reason is a personal one. During a period of some twenty years spent in almost daily contact with psychotic patients I have always been particularly interested in the strange ideas to which many of these patients give utterance and to which they adhere so tenaciously. Furthermore, with a view to discovering the why and wherefore of the delusions expressed, I have from time to time made studies of individual cases, recording as far as possible what they said and did during a period of several months. I therefore felt it suitable on such an occasion as this to choose as the topic for consideration one which had been made the subject of personal study, even though I was only too conscious of the fact that I had no very striking or original views to impart.

At the outset it would appear desirable to define a delusion, seeing that this is the subject of discourse. When an attempt is made to do so, however, many difficulties are encountered. An hallucination, which is a morbid symptom closely allied to a delusion, may be defined more readily. It is a perception without an object: a voice is heard when no one speaks, something is seen where nothing is. A delusion has been described as a false conception and persistent belief, unconquerable by reason, of what has no existence in fact. Now, while this definition is more or less adequate from the purely external point of view, it is inadequate from the point of view of psychiatry, as the delusions of the insane are assuredly based upon realities—realities the nature of which it must be the chief aim of the psychiatrist to discover. It does not necessarily follow, however, that because a belief is irrational and absurd it should be included in the category of delusions, because many persons who are regarded as normal, and who, indeed, are normal, entertain beliefs which to others appear totally incredible and unreasonable. It would thus appear well to renounce any attempt at defining a delusion, especially as this term can be used to convey a variety of meanings. Instead then of indulging in a meticulous discussion on the significance of a word, we may perhaps reach the essentials of our subject more quickly by utilising a delusion exhibited in a concrete case as a paradigm of delusions in general.

A man, aged 39, shortly after his marriage, developed a severe anxiety state. Though insisting he was quite well, he walked about in a state of restless agitation, picked himself, and could not concentrate. After a time the curious delusion emerged that he had the gift of flight, and this delusion has persisted ever since. At the present time his general intelligence and memory are perfectly normal, and he is able to converse sensibly, and even amusingly, upon any subject within the range of his knowledge and experience. He never seems really interested in a conversation, however, and he gives the impression that he finds it rather a nuisance, and has more important and pressing matters awaiting attention. The moment the conversation flags, the patient will mutter to himself, curse himself for a fool, and exhibit a number of jerky movements which betray an obvious difficulty in sitting still. He is a solitary man, and likes to hide away in some quiet spot, where he is free to indulge in a number of strange antics without the restraint or hindrance which the society of others imposes. He poises himself for flight, inflates his chest, makes all kinds of hissing noises, and persistently reproaches himself for failing to soar into the air. He dislikes talking about his delusions, and politely but firmly turns the conversation into other channels when the subject is introduced. I have spent many fruitless hours trying to penetrate below the surface. Often the patient shuts up like an oyster, and the usual responses when he consents to refer to his delusion consist of fragmentary and jerky sentences hissed out between his teeth. A typical instance is as follows: "I could do it if I tried—it *is* possible—I 'd better do it too—it comes from the brain—I could do it with my brain—my whole body will go through the air—it 's the only thing worth living for—I am a fool—why don't I do it?—it 's the only possible thing to do—I must be a man and do it."

Here then is a man who is impelled by a remorseless and persistent force within him to carry out a preposterously impossible task. Apart from his delusion he is an intelligent man, clear in his mind and free from confusion. It is true that he exhibits emotional indifference to matters which most people regard as important and interesting. Thus he is indifferent to the fact that he is in an asylum; he has no desire to return home; and he shuns social contacts. But this apparent affective apathy is due to the fact that he has only one thing to live for; all his energy is absorbed in a futile task, and he just wishes to be left alone in order to carry out his life work. All he knows is that he can fly if he makes sufficient effort, and that is what he wants to do.

As regards its actual content, there is nothing unique in the delusion of this patient. The human being has nourished the phantasy of flying throughout all ages. As Ricklin has pointed out, it appears repeatedly in the form of a wish-fulfilment in fairy stories. In these the wish to be able to fly is met by cloaks and enchanted birds as a means of transport; by a little bed with which one may be carried everywhere one wishes; or one is changed directly into a bird. Then it is a frequent theme in what Freud calls "typical dreams," by which is meant dreams in which almost everyone has dreamt in the same manner. Finally, it may be pointed out that some persons believe as part of their spiritualistic doctrine that, under certain conditions, not only may certain bodies move in the air without support, but that even the medium himself may be capable of floating in space under the influence of spiritual forces (levitation).

Now though we may regard as erroneous the belief that an individual infused with supernatural power may become so buoyant as to float in the air, we should assuredly not take the view that such a believer was, on account of his belief, suffering from a psychosis, any more than we should regard the superstitions of primitive races as evidence of abnormality. The (to us) fantastic beliefs of the native we recognise as normal for him, because we know that they are really not his own; they have not evolved from his own psyche, but are collective beliefs, and are part and parcel of his social heritage. Similarly with the belief in levitation: this is no more than one element of a widely accepted doctrinal complex which has many points of contact with religious beliefs generally. Thus while we may profoundly disagree with its tenets, they are invested with an atmosphere of familiarity, and for this reason, should they come under discussion, we feel at home with our subject, and are able to discover common grounds of agreement such as are essential in any disputations. We understand the language in which these beliefs are expressed; we recognise that they include concepts somewhat akin to those to which we ourselves may give our adherence ; we make concessions, and go as far as to admit that " there may be forces of which we have at present but little inkling," and perhaps allow that " there may be something in telepathy " ; and then maybe, feeling perhaps that we have conceded too much, we may proceed to enlarge upon collective hypnotism, the fallibility of human evidence, mediums who are known to have indulged in trickery, and so on.

The point I wish to emphasise is that such beliefs as those are no more than variants of beliefs common to all people and all times. The individual acquires them from his social *milieu.* The notion of individuality on which we are liable to pride ourselves is, indeed, largely an illusion. Our language, concepts, beliefs, our behaviour, patterns, tastes, opinions, and even our feelings, which are largely conventionalised, are derived from our social environment. The difference between individuals are infinite in their variety, but these differences are fundamental and unconscious ; they do not depend so much upon variations in opinions and beliefs, but upon natural abilities and temperament. Briefly, they are determined by organic factors which are inherent, and not upon the accidents of environment. Every parent and educationist knows that there is some intangible and indefinable factor at work in a child which exerts a constant influence upon its reaction to experience—a factor which education and training are powerless to alter.

These few observations enable a contrast to be drawn between the irrational beliefs of normal persons and the delusions of the insane. In the case described the delusion is clearly an irrelevant intrusion into the psychic life. It bears no relation to pre-existing beliefs, to personal interests, or to problems upon which the patient may have pondered. He will not so much as lift his head to observe an aeroplane, and it is evident that no connection exists in his mind between *that* flying and *his* flying. The delusion is inspired from within, and not suggested from without, and it clearly belongs to an altogether different category of belief than one derived from the social *milieu.* In his fascinating chapter on the perception of reality William James wrote as follows: "Each thinker . . . has dominant habits of attention; and these practically elect from among the various worlds some to be for him the world of ultimate realities. From this world's objects he does not appeal. Whatever positively contradicts them must go into another world or die. The horse, *e.g.*, may have wings to its heart's content, so long as it does not pretend to be the real world's horse—*that* horse is absolutely wingless. For most men . . . the 'things of sense' hold this prerogative position, and are the absolutely real world's nucleus. Other things, to be sure, may be real for this man or for that—things of science, abstract moral relations, things of Christian theology, or what not. But even for the special man, these things are real with a less real reality than that of the things of sense. They are taken less seriously; and the very utmost that can be said for anyone's belief in them is that it is as strong as his 'belief in his own senses.'"

In the deluded psychotic there may actually be a definite reversal of James' "reality formula," so that the external world which impinges upon his senses becomes for the patient a less real reality than the delusion to which he adheres. In every case the insane belief has a reality-value at least equal to that of the material world around him. One immediately feels this in conversing with a patient. His attitude is quite different to that of a normal person in relation to (say) his religious beliefs, for however sincerely these may be believed in, it is at least recognised that their validity may be open to question. Moments of doubt are also apt to arise, when such beliefs need to be reinforced by acts of faith. It is otherwise with the deluded person; he does not merely believe—he *knows*, just as we know that an object before our eyes has a real existence. The reason for this is not far to seek; the delusion is the outward and visible sign of an inward reality—the symbolic expression of endogenous or organic disturbances. The delusion thus differs from superstitions or erroneous beliefs in that it does not originate from the social *milieu*, but from the organism itself; it is invested with the same "reality-feeling" as perceptions stimulated from without, because it is itself an intuitive perception of organic stimulations from within.

At this juncture it is necessary to point out that the living organism is not quite the same thing to the psychiatrist as it is to the general physician. The latter, as such, is concerned only with disorders of the physiological functions, whereas the latter is concerned with disturbances of behaviour, that is to say with disturbances of the reaction of the organism as a whole to the environment. Now a complete knowledge of the physiological state of the organism of an individual is insufficient in itself to account for his behaviour at any given moment. This is determined by a complex of mutually interrelated factors, amongst which are his physical state at the time, the whole of his past experience, his personality-type, and his ancestral history. All these factors play their part in the reaction of an individual, whether abnormal or normal, to life. Thus the organism to the psychiatrist is not merely a physical organism, it is an organism "impregnated with psychism"—one in which both the individual and racial experiences exert an active influence at every moment. As White observes, we must consider the individual as possessed of an unconscious which contains the psychic precipitate of millions of years of experience (mneme), as the body contains that experience laid down in structure. With this brief digression we will return to the problem of delusions.

I must now refer to a highly-important clinical characteristic exhibited by the delusional psychotic, this being that he is quite unconscious of the source of his delusion. Though we know that his morbid thoughts and feelings are the conscious symbols of disturbances in his organic life, the patient is quite ignorant of this fact, and he would strenuously combat any such suggestion. There is in this respect a marked difference in the attitude of a patient towards physical and mental illness respectively. Both types of malady are the expression of disturbances of the organism; but in the one case the patient realises he is ill and can more or less localise and describe his sufferings, while in the other, in most instances, the patient has no sense of illness, and he is unable to furnish any information as to the nature or locality of the stimuli responsible for the morbid psychic products which surge into his conscious life. Thus the morbid state of the organism is not represented in the mind of the patient as physical suffering, but as depression, unappeasable anxiety, a feeling of guilt, delusions of omnipotence or persecution, or in the form of visual, auditory, or conæsthetic hallucinations. This is clearly seen in the case cited above. The patient does not feel himself to be physically ill; on the contrary, he believes himself to be the possessor of a unique gift—the gift of omnipotence, indeed, since at the back of the delusion of flying are two other beliefs, the first being that he is the only man who will ever be able to fly, and the second, that when he succeeds in so doing he will be endowed with universal knowledge.

What then is really wrong with the organism in these chronic delusional patients? In regard to this matter, the psychiatrist, it must be confessed, is somewhat in the same position as his patient, for he also is unable to localise the seat of the malady, and a most exhaustive physical examination is often entirely negative. The body of the patient appears normal, and he often lives to a ripe old age. Formerly it was supposed that the secrets of disordered personality could be discovered by researches confined to the brain, but the clinical characteristics of many chronic delusional cases do not suggest the existence of structural lesions in the cerebral cortex, such as are discoverable in general paralysis in its advanced stages or senile dementia. In many of these deluded patients the memory and general intelligence are unaffected, even though they may nourish fantastic delusions and exhibit extreme abnormalities of conduct. Thus a patient, who had been for several years inaccessible, negativistic and mute, suddenly began to write poetry, in which she gave a detailed account of the happenings in her ward during the years of her illness. The accuracy of her memory and the keenness of her perception were nothing short of remarkable. Thus it is not the intellect or judgment which are primarily responsible for delusional states, and most psychiatrists now recognise that these have an emotional rather than an intellectual basis. Behind the thinking, logical being, whose development coincides with the development of the neuro-cerebral apparatus, there exists a non-logical, affective being which, as Hesnard says, is a psychic expansion of the life of tropisms, reflexes, and organic tendencies, themselves the basis of instinct. It is from disturbances of this organic-instinctive life that delusions appear to originate.

I have suggested elsewhere that thee volution of a delusion is often similar in nature to the unfolding of an instinct. Clear examples of pure instinct are observed in activities concerned with reproduction and the rearing of the young. Birds mate, build a nest, lay eggs, sit on the eggs, feed their young, and care for them as long as is necessary. An impulse from within impels the bird to perform this series of extremely complex reactions, though it can obviously have no knowledge of why it is behaving thus, or any prevision of the ends to be attained. The development of the delusion in our patient closely resembles the birth of an instinct in animals. At first he experienced a sense of anxiety, discomfort and tension—the consciousness of changes in the organic life for which he was unable to account. Then the delusion crystallised out, and he became infused with a new sense of power and purpose as if he had made a great discovery. He made no attempt to justify his belief; it was an "*a priori* synthesis of the most perfect sort," and any suggestions casting doubt upon the reality of his unique and ineffable gift were to him as mere flippancies. Like an instinctive craving a delusion develops, wells up, or emerges of itself, and the patient himself is powerless to prevent what is taking place. The delusion unfolds itself; it is "hatched-out" of the organism, as it were.

The content of a psychosis often reveals the existence of a disturbance in the instinctive life of the organism. Thus visual, auditory and conæsthetic hallucinations more often than not have a definitely sexual colouring. As an instance may be cited the case of a patient who, having served the State and led a life of high endeavour, after a period in which he imagined himself to be the victim of blackmail, becomes haunted by visual hallucinations of a grossly homosexual type; and then a formless black figure materialises and follows him wherever he goes. Then we know how closely connected is the development of the psychoses, with the ripening or decay of the sexual life, or with betrothals, marriage and childbirth. In other cases the origin and significance of the delusion are veiled in obscurity. The delusion of flying is a case in point. That sexual components enter into its causation I do not doubt, but I have always felt reluctant to give a strictly sexual interpretation to a symbol unless the relationship was quite evident. Let us therefore here content ourselves by saying that to the patient (for undiscovered reasons) it represented a feeling of omnipotence with which his ego had become inflated.

Since a study of the psychology of the biogenetic psychoses suggests that their symptomatology is the outcome of disturbances in the organic instinctive life, it is not to be expected that the psychiatrist will be able to localise the seat of these maladies in any single organ, not even the nervous system, or to describe the exact nature of the physiological changes which may be presumed to be taking place. Furthermore, I feel very doubtful if the delusions and hallucinations of the insane can be psychologically localised as Freud has endeavoured to do. We know that the delusions of the schizophrenic alway range with persistence around the region of the genital organs, but, as Kretschmer observes, we must neither separate the total affectivity from the sexual impulse, nor account for it entirely in terms of the sexual impulse.

In an interesting monograph upon the psycho-physiology of hunger Turro points out that the feeling of hunger or thirst has for its cause the impoverishment of the internal *milieu*, and in consequence the distress of the cells of the organism. The internal *milieu* is not an inexhaustible storehouse; its supplies have to be continually renewed, and the time arrives when either the quality or the quantity of the internal resources is insufficient to supply the cellular avidities. It is then that what we call hunger is consciously felt. There is here a difference between vegetable and animal behaviour. In the former the conative impulses or strivings—here mainly concerned with assimilating the chemical material necessary for the metabolism of vital existence—do not take the form of seeking movements in relation to the environment, but, as Briffault well puts it, "of a continuous appetence continuously satisfied." The plant bathes in its food, it does not search for it and procure it. It is otherwise in animal life. A moment occurs when its organism can no more of itself supply the internal *milieu* with what is necessary to nourish its cells ; the unique means then of repairing the deficit is to seek for and to reincorporate within itself the elements which it lacks. "At this stage," to quote Turro, "this psychic tendency, which impels to seek without what is lacking within, is what constitutes the sensation of hunger—an echo of physiological distress, and at the same time the dawn of psychic life."

The bio-psychic processes involved in delusional formation bear a close resemblance to those occurring in the awakening of hunger. Just as hunger is the echo of normal physiological distress, so a delusion is the echo of morbid physiological distress—the symbol of diffuse and unlocalisable changes occurring in the depths of the organic life. Hunger does not originate in the brain, but in the depleted cells of the whole organism. It is thus, also, with a delusion ; it is the conscious symbol of a morbid state of functioning of the whole organism. As Mott, whose conclusions were based upon exhaustive researches on the pathology of dementia præcox, suggested, there is probably a hypofunction of the whole of the bodily tissues, specially of the reproductive and endocrine systems and associated with deficient oxidation processes. I would like to refer also to the important investigations carried out by Miss Robertson under the direction of Dr. Golla at the Maudsley Hospital on the vaso-motor reactions in mental disorder, with special reference to hæmoclastic crisis. This crisis is characterised by leucopenia, fall of blood-pressure, inversion of the leucocytic formula, hypercoagulability of the blood, and diminution of the refractive index of the serum. To demonstrate the presence of the hæmoclastic crisis, 200 grm. of milk are administered to a subject who has fasted for several hours. The leucocytes and differential leucocytes and blood-pressure are noted before the meal is taken, and again at intervals of twenty minutes afterwards. In the normal subject the blood-pressure either remains unaltered or tends to rise. The findings of this research need not be here detailed. For the present purpose it suffices to note that the hæmoclastic crisis occurred in 94 per cent. of dementia præcox patients, in 85 per cent. of melancholias, and in 75 per cent. of chronic mania cases.

Thus associated with the mental disturbances of these patients are disturbances in their organic life. As in the case of hunger, the total reality is not revealed by exclusive reference to either the unconscious biological or the conscious psychological processes. The total reality is a bio-psychic process. We are dealing with the organism as a unity, one and indivisible. It is thus with a psychosis, where we have a morbid state of the organism overtly manifested by a morbid state of mind. As to the causes of this endogenous morbidity, various opinions have been expressed. I recently had the advantage of a conversation with a distinguished continental psycho-analyst, who expressed the view that dementia præcox might, with further knowledge and investigation, prove to be psychogenetic in origin. I take it that the psychogenetic theory in pure form means that a normally constituted individual might be so inhibited from direct or sublimated expression of his sexual cravings (life-force) by defects in his upbringing and life situations, that these cravings eventually find an outlet in consciousness in the form of delusions and hallucinations. I wish most sincerely that I could accept such a view; if it covered the facts it is evident that measures might be taken along educational lines which should do much to diminish the incidence of insanity in the community. The psychogenetic factor must, of course, not be ignored in considering the pathogenesis of the biogenetic psychoses. Since the past experience of an individual exerts an active influence upon his present behaviour, it is clear that we cannot exclude the environment as a factor in the production of the psychoses. If the forces of life are pent-up by an environment affording them an inadequate outlet, a chronic state of bio-psychic tension may result—a state of neuro-vegetative and humoral disequilibrium on the one hand, and of emotional disquietude on the other. There can be but little doubt that neuroses may be thus created or that these can be cured by psycho-analytic or other psycho-therapeutic measures. As regards the biogenetic psychoses, however, I cannot but feel that, in respect to causation, the accent must be placed upon the organic rather than upon the psychogenetic factor. The subject of dementia præcox is not so much the victim of external circumstances as he is of an inadequate bio-psychic (organic-instinctive) constitution. To put the matter crudely, in the psychoses the abnormal psychic manifestations appear to be the expression of *diseased* instincts, rather than, as purely psychogenetic theories would seem to imply, of *distorted* instincts. Personally I believe that the divergence of opinion between the psychogenetic and the biogenetic schools of thought is a purely artificial one. It is not a question of whether a given abnormal mental state is due to the external pressure of life or to an organic disease, but rather a question as to *how much* it is due to one or the other.

In the normal person the rhythm of his organic life goes on in the silence of the unconscious ; at the most it constitutes a dim background to clear consciousness. At times, of course, physiological needs attract attention, but it is essentially in disease that organic processes become insistently articulate. Thus in pneumonia, for instance, the patient becomes painfully aware of the fact that all is not right with his organism. A similar state of affairs obtains in the psychoses, where organic disturbances become articulate in the form of delusions and hallucinations. The patient becomes conscious of what should be unconscious, namely, the functioning of his own organic being. The majority of these manic-depressive or schizophrenic patients are endowed with a constitutional blemish of their bio-psychic equilibrium. In spite of viscera usually normal, and a physique often, but by no means always, normal, in spite, also, of an intelligence sometimes above the average, they are unhappily the victims of an organic life, anarchical and imperious, the generator of powerful and inco-ordinated emotional tendencies. Such individuals are peculiarly liable, as a result of fatigue, psychic trauma, some infection, focal sepsis (an etiological factor which has been made the subject of some interesting observations by Bristol clinicians), or the normal physiological stresses, to develop definite psychoses. The pathogenesis of the delusional development in such cases has been well described by Hesnard. The following is a brief resume of his views. Originating at the heart of the undifferentiated organic; diffuse and unlocalisable expression of a disorder of the instinctive life—the affective urge of the psychopathies is not transmitted to the psychic organ by the ordinary physiological route of general (conæsthetic) sensation, as are at least partially the organic appetites, but by different and extremely diffuse paths which represent the roots of this organ in the organic life. These are not made to admit the conscious symbols of sensation, but only of "feeling." In consequence of this, the affective urge is experienced, not as an illness or a pain, but as euphoria, anxiety, excitement or depression, which effects are quickly symbolised into objective imagery. Thus a psychotic is suffering from a malady of a particular kind. His illness changes so intimately, and in a manner so diffuse, those organic functions upon which the integrity of his personality depends, that in place of perceiving his true malady he perceives only a sense of power, a strange being within him, or an image (hallucination) which he localises outside himself.

This formulation appears to cover the facts. Many years' clinical experience has led me to the view that, in their ultimate analysis, the psychoses are no more than obscure forms of organic, though not necessarily incurable, disease. Not infrequently we have the happiness of observing the delusions of our patients melt away, so that complete recovery ensues. When this happens, we may suppose that a bio-psychic adjustment occurs, similar to that occurring in (say) pneumonia, where the psychic manifestations of pain and distress melt away as the pulmonary disorder subsides. Such a view is not necessarily antagonistic to a psychological theory of causation. The two are complementary rather than contradictory; they envisage different aspects of the same reality. I would point out the following, however: the study of the instinctive behaviour of animals deprived of food has been responsible for important additions to our psychological knowledge; from the strictly practical point of view, however, we know that the disturbance in their behaviour can only be cured by supplying their organism with what it lacks. It may be that here we have a hint as to the kind of treatment which may some day prove to be the most effective in the cure of the constitutional psychoses. We have the example of myxœdematous insanity upon which to reflect, and from which to gain encouragement for unremitting research.

From what has been said, it is evident that when a patient expresses some grotesque delusion, he is, in a sense, asserting what is true. He has, indeed, more right on his side than has one who would persuade him to the contrary, for his delusion is but the symbol of organic actualities. There is a morbid stir in the organism which stimulates into activity latent instinctive tendencies, and also, as Storch has shown, archaic modes of thought which lie deeply buried in the organic unconscious. Delusional thought is "organic" thought which evolves like a dream, unhampered by the cramping influence of external reality and the laws of time and space. As Blondel observes, "The morbid consciousness expresses itself outside the laws of social consciousness by mental processes, not only pre-rational, but pre-normal, which reveal the *individuel pur.*" French psychiatrists apply the term *aliéné* to the psychotic patient, and as regards his delusions he is definitely alienated from social life.

In so far as the social consciousness of our patients remains intact, we may share their joys and sorrows, and they ours ; but in respect to their delusions, we can have no mutual spiritual communion. Something altogether unique is created in a psychosis ; the mind is invaded by morbid mental growths which are unanalysable, primordial, and, to the normal mind, incomprehensible. A delusion is a strictly individual psychic product, and often the only elements in it which are derived from the social *milieu* of the patient are the language symbols he employs in the vain endeavour to make himself understood.

